# From Broken Windows to Perceived Routine Activities: Examining Impacts of Environmental Interventions on Perceived Safety of Urban Alleys

**DOI:** 10.3389/fpsyg.2018.02450

**Published:** 2018-12-04

**Authors:** Bin Jiang, Cecilia Nga Sze Mak, Hua Zhong, Linda Larsen, Christopher John Webster

**Affiliations:** ^1^Department of Architecture, Division of Landscape Architecture, The University of Hong Kong, Pokfulam, Hong Kong; ^2^Virtual Reality Lab of Urban Environments and Human Health, Faculty of Architecture, The University of Hong Kong, Pokfulam, Hong Kong; ^3^Department of Sociology, The Chinese University of Hong Kong, Shatin, Hong Kong; ^4^Department of Landscape Architecture, University of Illinois, Urbana-Champaign, IL, United States

**Keywords:** high-density city, perceived safety, broken windows theory, routine activities theory, vegetation, urban function, environmental intervention, urban alley

## Abstract

In high-density cities around the world, alleys are common but neglected spaces that are perceived as unsafe. While cities have invested resources in environmental interventions to improve safety in urban allies, it is not clear how these interventions impact perceived safety. We review two important criminology theories that discuss the environmental and social factors that lead to crime: the Broken Windows Theory and the Routine Activity Theory. We argue that these theories can also be used to explain safety perceptions of urban environments, and then develop urban alley interventions based on these theories. We test people's perceived safety of these interventions through a photograph survey. Results show that all interventions yielded higher perceived safety than existing alley scenes. Interventions based on the Broken Windows Theory (cleaning or vegetation interventions) yielded only modest improvements in perceived safety, while interventions based on the Routine Activity Theory (urban function interventions) yielded higher ratings. Our findings question the dominant use of the Broken Windows Theory in environmental interventions to promote perceived safety and argue for a more effective approach: urban function interventions inspired by the Routine Activity Theory.

## Introduction

The urban alley^1^ is a common but often feared urban space in high-density cities around the world. Urban alleys are often underutilized, underdeveloped, and deteriorating spaces in cities (Seymour et al., 2010, p. 380). They are seen as hot spots for crimes such as robbery, prostitution, drug dealing, rape, or homicide (Seymour et al., 2010; Zambito, 2010; Mok, 2012; Rucki, 2014). Frequent news reports of vicious, violent crimes in urban alleys contribute to this perception. In New York City in 2010, for instance, a young woman was dragged into a urban alley by a man, brutally raped and killed (Zambito, 2010). In a central urban area in Hong Kong, local media reported 60 serious crimes in urban alleys from January 2014 to September 2015. Similar media reports can be found throughout the world. Even when actual crime rates in alleys are low, people often perceive them as unsafe, and these perceptions cause them to avoid or modify their behavior in alleys. In cities like New York, Chicago, London, Hong Kong, and Shanghai, cities with hundreds or even thousands of urban alleys, creating urban alleys people perceive as safe is a pressing issue.

Urban alleys have rarely been examined in empirical studies in the field of environmental design, but previous studies in other urban contexts suggest that environmental interventions can improve the safety of public places (either perceived or actual safety) (Yang et al., 2013; Branas et al., 2018). Some cities have attempted to revitalize urban alleys through environmental interventions to enhance citizens' health and well-being and to promote economic development and neighborhood vitality. However, it is unclear whether these efforts make citizens feel safe (Daley, 2007; Nathanson and Emmet, 2007; Newell et al., 2013).

In addition, environmental designs and interventions should be grounded in established psychological and criminology theories that have been empirically tested. The theory that is most often associated with environmental interventions to improve actual or perceived safety is the Broken Windows Theory, but do urban alley interventions based on this theory actually improve perceived safety? We explore a second important criminology theory, the Routine Activities Theory, and argue that it can be used to explore perceived safety of environments as well. We test interventions based on both theories to understand how they impact perceived safety. Without understanding people's safety perceptions of different urban alley interventions, we risk wasting resources redesigning urban alleys in ways that do not help people feel safe. If people perceive these interventions as unsafe, they will likely be underutilized, underdeveloped, and may fall into disrepair.

We begin by describing the theory and literature on environmental design and safety perceptions and use this theory to justify the urban alley interventions we develop. Next, using Hong Kong as the setting for our research, we conduct a photo questionnaire exploring people's safety perceptions of existing urban alley conditions and of a variety of urban alley design interventions, including cleaning and adding green landscapes and urban functions. We discuss the results and make design recommendations for urban planners and landscape designers seeking to transform urban alleys in high-density cities into safe and vital spaces.

### Urban Alleys and Perceived Safety: Theoretical Explanations

Why do people perceive urban alleys as unsafe, and what environmental and social characteristics influence perceived safety? Two relevant theories provide a rationale for this study: the *Broken Windows Theory (BWT)* (Wilson and Kelling, 1982) and the *Routine Activity Theory (RAT)* (Cohen and Felson, 1979). These two theories have been widely used to guide practical policies for improving safety among citizens and for reducing crime (Rountree and Kenneth, 1996; Skogan, 2008).

#### Broken Windows Theory (BWT)

Wilson and Kelling's Broken Windows Theory (1982) explores how environmental disorder can shape crime levels and people's perceptions of an environment. Orderliness (following social norms) delivers the subjective social meaning that the community is under control and criminals are in check, deterring criminal activity. On the other hand, disorder delivers subjective social clues that the community is not under control. Disorder has been shown to induce deviance/crime and lead average citizens to withdraw, causing neighborhoods to decay and quality of life to decline (Keizer et al., 2008; O'Brien, 2015).

Order-maintenance policies are often referred to as an important crime deterrence pathway.

The BWT also seems to suggest that disorder influences people's perceptions of safety. Disorder is the major cause of people's fear of an environment, more than actual crime rates or victimization risks (Skogan, 2012; Hinkle and Yang, 2014). People's perceptions of disorder are mainly influenced by objective signs, such as graffiti, poorly maintained landscapes, litter, vandalism, poor lighting, or painted advertisements in a place (Osgood et al., 1996; Sampson and Raudenbush, 2004; Seymour et al., 2010; Troy et al., 2016).

Prior studies have offered psycho-social explanations of why disorder is associated with fear of crime or perceived safety. Disorder in urban alleys sends the message that nobody cares for the place, which might make people feel threatened, while reducing the perceived cost of committing a crime for potential offenders (Zimbardo, 1973; Kotabe, 2014). Humans experience primitive negative emotions when they feel threatened (e.g., feeling distressed, powerless and unsafe). Perceptions of disorder also influence individuals' socially-constructed judgments about local safety (Hinkle and Yang, 2014; O'Brien et al., 2014). For instance, people might interpret piles of clutter or waste in urban alleys as hiding places for potential offenders and indications of criminal activity (Wilson and Kelling, 1982; Sampson and Raudenbush, 2004).

The relationship between perceived safety and disorder is cyclical: disorder decreases people's perceived safety; lower perceived safety leads to avoidance of a space; and avoidance leads to further disorder (Foster et al., 2014). Disorder can lead to an “epidemic” of crime and neglect in a community (Welsh et al., 2015). BWT suggests that to break this cycle, environmental signs of disorder need to be removed and signs of order need to be added, which will deliver a favorable social meaning and improve the safety perceptions of the community. Environmental interventions based on BWT mainly focus on changing the physical appearance of a space to deliver a favorable social meaning.

Although BWT has been embraced by many researchers and practitioners, many other studies question the efficacy of the Broken Windows Theory. Some researchers argue that disorder is not a decisive indicator of crime risk, while the social conditions of the environment are (Taylor, 2001). For example, Gau and Pratt (2010) point out that disorder, crime, and perceptions of each are complex. They dispute the notion that disorder causes fear of crime and actual crime, and assert that socio-structural conditions should be considered as the main driver of deviant behavior and crime. O'Brien and Sampson (2015) found failure of social cohesion to be more closely associated with violent crime than public physical disorder.

#### Routine Activity Theory (RAT)

In contrast to BWT, the Routine Activity Theory focuses on the convergence of three key social factors that influence crime occurrence: a likely offender, a suitable target, and the absence of a capable guardian (Cohen and Felson, 1979). To drive away motivated offenders, protect potential victims, and promote guardianship, RAT suggests that spaces need legitimate routine activities—“recurrent and prevalent activities (such as grocery shopping and commuting) which provide for basic population and individual needs” (Cohen and Felson, 1979, p. 593).

In the past few decades, scholars have translated this macro-level theory to the micro (individual) level (Tittle, 1995; Osgood et al., 1996; Pratt and Turanovic, 2016). These scholars point out important limitations of Cohen and Felson's original theory and have developed it in many ways. First, Cohen and Felson (1979) didn't clarify what kinds of routine activities decrease the likelihood of crime, nor the social conditions at specific urban spaces that might lead to a higher risk of crime (Tittle, 1995). There are many different kinds of routine activities which would likely have differing impacts on crime. The individual's lifestyle should also be a key factor. Second, motivated offenders, vulnerable victims, or capable guardians are not equally or randomly distributed in our society. More recent critics note that “probability” plays an important role: Different social structural conditions in an environment cause people to perceive risks differently when they participate in routine activities (Pratt and Turanovic, 2016). Based on these criticisms, scholars have sought to make RAT a more comprehensive and valid theoretical framework.

Both the original and contemporary RAT mainly explore factors that lead to actual deviant behaviors or crimes, not the factors that influence whether places are perceived as safe. Nevertheless, we suggest that this theory can also be applied to investigate perceived safety of environmental interventions. There is evidence to suggest that spaces which encourage routine activities not only have lower crime rates, but are also perceived as safer (Branas et al., 2018). Another study has identified the positive relationship among legitimate routine activities, increased guardianship, and perceived safety (Meghan et al., 2013). People in protected spaces with increased guardianship have lower uncertainty, receive more information, and feel more powerful, leading to higher levels of perceived safety (Nasar and Fisher, 1993; Nasar and Bokharaei, 2017). Empirical evidence suggests that subjective feelings of safety and insecurity are highly situational and related to individuals' routine daily activities (Doran and Burgess, 2012; Solymosi et al., 2015).

Applying RAT to environmental design research about perceived safety raises an important question: If people's perceptions of safety are influenced by the routine activities that are likely to take place in an environment, can people ascertain routine activities from photographs, image simulations, or even actual encounters with a place? Researchers argue that people have an innate ability to understand a place and estimate risk of victimization through a quick visual perception (Kaplan et al., 1998; O'Brien and Wilson, 2011). Clues in the visual environment help people make this judgment. Specific urban functions or features help people infer the kinds of routine activities that will occur. A street with many bars, for instance, will elicit more drinking behaviors, while a street with museums, theaters, or bookstores will elicit more cultural activities. Through this inference, they can quickly make a judgment about how safe it is. Places with intense, diverse, prolonged, and legitimate routine activities are likely to be perceived as more safe. It is our assertion that people will be able to infer the routine activities of an urban alley through visual clues shown in photographs, and this inference will influence their assessment of an alley's safety.

### Environmental Interventions Based on Theories

Below we describe potential interventions based on these theories and literature that addresses the effectiveness of these kinds of interventions in increasing safety, both actual and perceived.

#### Removal of Disorder

Broken Windows Theory (BWT) suggests that the disorderly appearance of a place, including the presence of physical and social incivilities, significantly impacts perceived safety and crime rates. Interventions grounded in this theory seek to clear the disorder to alter people's perceptions and behavior. Removing disorder would create a new visual appearance but would be less likely to provide a new social dynamic for that place (Welsh et al., 2015).

#### Vegetation

In addition to removing signs of disorder, urban professionals often add vegetation in urban spaces to promote safety. Also grounded in the BWT, this intervention improves perceived safety by altering the appearance of the place to show a more inviting social image. Studies suggest well-maintained vegetation can act as a territorial marker or a cue that the space is cared for, thus discouraging deviance and crime (Kuo et al., 1998; Troy et al., 2016). The presence of vibrant, orderly vegetation is a strong indicator of human management while a barren space is often perceived as neglected land (Troy et al., 2016).

A handful of studies reported that the presence of vegetation also promotes safety by encouraging greater use of public space, thereby providing greater social supervision (Kuo and Sullivan, 2001; Branas et al., 2011, 2018; Donovan and Prestemon, 2012). In some circumstances, adding vegetation can change the function of the space, inviting people to participate in legitimate routine activities. A spacious barren area can be transformed into a public green space for a wide range of recreational activities. For example, researchers exploring vegetation in a Chicago public housing complex reported that areas with greater vegetation had more users (Coley et al., 1997). However, in a narrow urban alley, there would be less space for recreation, and adding a small dose of vegetation would more likely transform the appearance rather than the actual function of the space.

Other findings suggest that added vegetation does not always make people feel safer. Some studies indicate that vegetation makes a space seem less safe if it is naturalistic (Yang et al., 2013), is poorly maintained (Jansson et al., 2013), contains too much undergrowth (Jansson et al., 2013), or creates a visual obstacle or conceals things (Madge, 1997). Another study reported the presence of short trees in front of or adjacent to house properties is positively associated with neighborhood crime rates (Troy et al., 2016). Together, these findings suggest vegetation can make a positive impact on perceived or actual safety if it is well designed and maintained.

#### Urban Functions

RAT emphasizes improving the safety of a space by changing the function of an urban space to create a healthy social structural setting that supports routine activities and promotes a stronger social dynamic with greater informal supervision. The social dynamic and supervision should be more intensive, diverse, enduring, and have a stronger functional connection with other places, residents, or visitors in close proximity. Urban alleys are mostly seen as passageways, but they can be transformed to become destinations for daily recreational, entertainment, shopping, exercise, or other routine activities. Jacobs (1961) emphasizes that parks can enhance people's perceived safety because they embrace urban activities and increase the number of eyes on the street (more capable guardians). Spaces with public amenities attract regular citizens for their daily activities. People can participate in necessary, optional, or social activities, which enhance supervision and increase people's perceived safety. In one of the few studies evaluating recent urban alley greening strategies, people preferred transformations that highlight not only cleanliness but also functions and recreational opportunities (Seymour et al., 2010).

Not all urban functions and amenities may increase perceived safety. Prior studies have shown some urban amenities may encourage risky activities and lead to rising crime rates. For example, Furr-Holden et al. (2011) found more abandoned buildings were associated with lower social control and then adolescents' higher rate of drug use because those buildings provided spaces for drug dealing and usage. Roncek and Pamela (1991) found that the number of taverns on 4,400 blocks of Cleveland was significantly associated with criminal activity because alcohol use may lead to aggressive behaviors (more motivated offenders) and individuals visiting the taverns may carry a lot of cash (more attractive victims).

### Critical Knowledge Gaps and Research Questions

This study addresses two important knowledge gaps. First, we ask to what extent interventions grounded in the Broken Windows and Routine Activity Theories impact perceived safety. The Broken Windows Theory has long been the dominant paradigm to explore the perceived safety of an environment. While studies have explored the relationship between visually perceived disorder and perceived safety, few studies have examined how alterations of urban functions (land uses) in an environment change the structural setting of routine activities, and how these interventions impact perceived safety. We compare interventions inspired by these two theories to test how well they improve perceived safety and make recommendations about how these criminology theories should inform environmental psychology and environmental design, and vice versa.

Second, although some studies have explored the impact of a variety of environmental interventions on people's perceived safety in other urban spaces, few studies have investigated their effect in urban alleys in high-density urban areas. We do not know whether, to what extent, and why these interventions impact perceived safety in this common but often neglected urban space.

To fill these important knowledge gaps, we use a photo-simulation survey to investigate how different interventions in urban alleys influence citizens' perceived safety. We raise two research questions: (1). How do citizens perceive the safety of existing disorderly urban alleys? (2). To what extent do various categories of urban alley design interventions based on BWT and RAT influence citizens' perceived safety? Through answering these questions, we aim to provide not only empirical evidence but also a theoretical foundation to create safer urban alleys and other urban spaces.

## Methods

### Background of Site District

**Figure 1:**
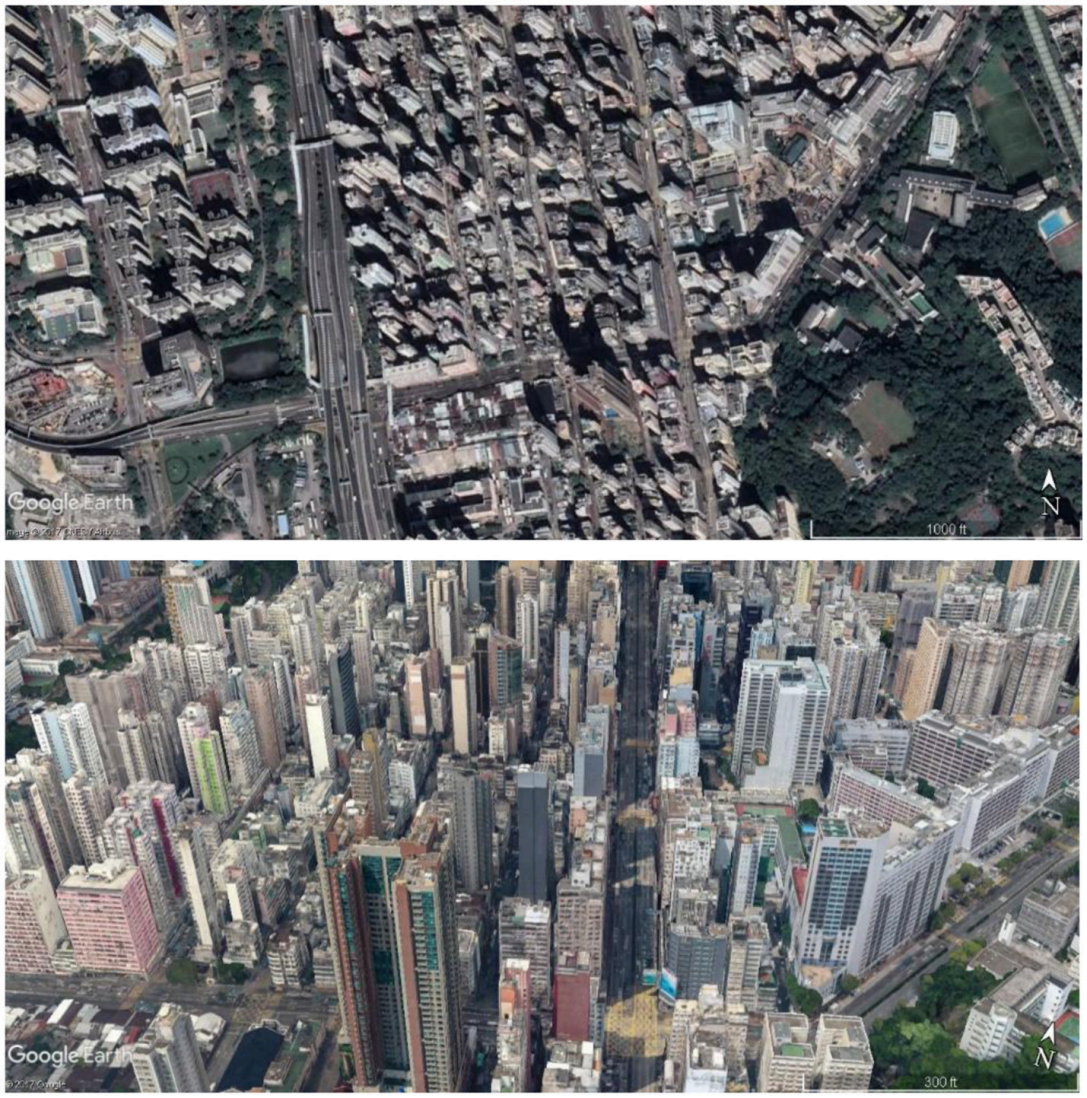
We investigated urban alleys in the Yau Tsim Mong District in the central area of Hong Kong City (Image: Google Earth Pro).

We selected urban alley sites in the Yau Tsim Mong District, located on the southern part of the Kowloon peninsula in Hong Kong (Figure 1). This district lies in the region in Hong Kong with the highest crime rate (Hong Kong Police Force, 2014). Since it was one of the earliest developed harbor and commercial areas in Hong Kong, it contains significant socio-economic and cultural diversity. The area is dominated by high-rise residential and commercial buildings. Most buildings have been built or rebuilt since the middle of the twentieth century with modern architectural styles. According to the 2011 population census, 307,878 individuals reside in this seven square kilometer district (54% of residents are females), suggesting it is an extremely crowded urban area. A variety of illegal businesses thrive in this district, including gambling, prostitution, selling counterfeit or fake goods, and drug dealing. The district is familiar to Hong Kong residents because of the advanced public transportation system connecting all city districts. It is representative of the complicated mixed zoning of Hong Kong and is not distinctly different from other urban areas in Hong Kong.

### Site Selection

Three investigators identified 58 urban alleys in the core area of the district through a street-view survey through Google Maps. One investigator then visited the sites and took panoramic photographs of each urban alley. The investigator stood by the sidewalk of the main street and faced the entry of the alley and then took an eye-level panoramic photograph. The distance between the investigator and entry was approximately two meters. The investigator set the angle of the viewshed at approximately 120° to include not only the urban alley space but also the street façades on both sides of the urban alley entry.

**Figure 2:**
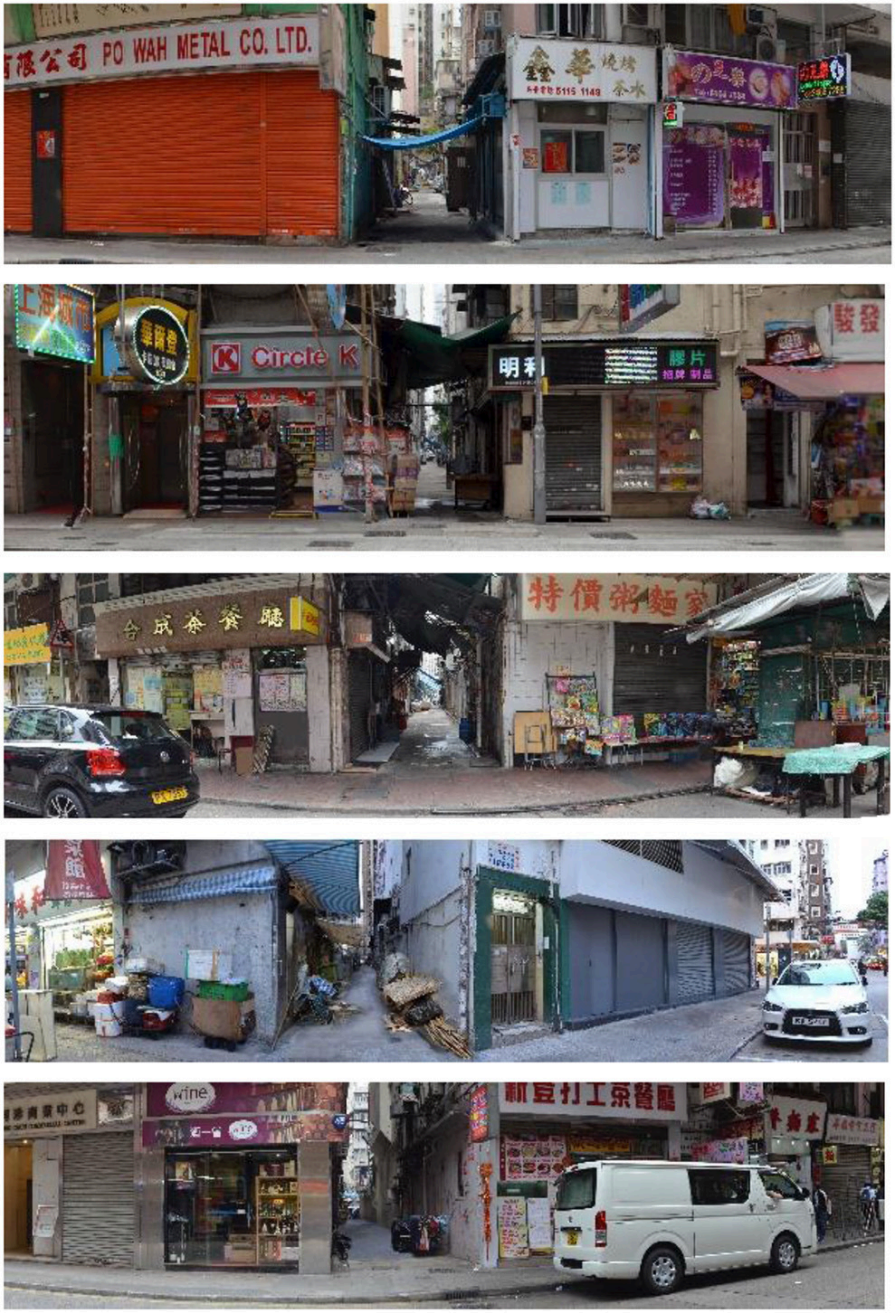
Three experts selected five scenes of urban alleys for this research through a restrictive procedure.

**Figure 3:**
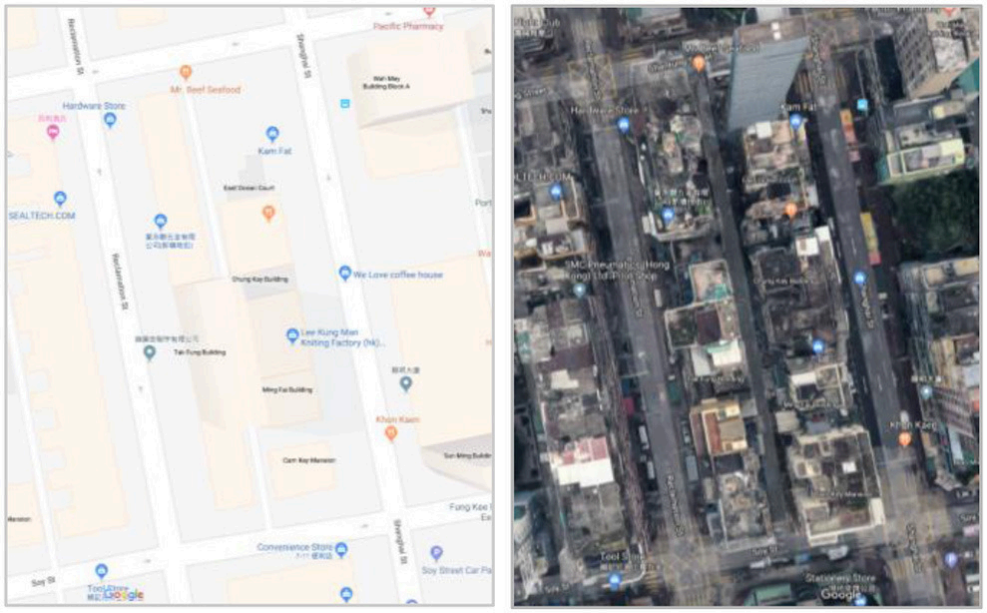
An urban alley site between the Reclamation Street and the Shanghai Street in Hong Kong. Source: Google Map.

Photographs of 58 sites were evaluated by three investigators with expertise in crime prevention through environmental design (CPTED) and post-graduate education in Landscape Architecture (Cozens and Love, 2015). A series of criteria was used to remove sites containing unusual objects or information, including unique looking buildings, unusual or special vegetation, the presence of birds or animals, unusual lighting conditions, unusual architectural decorations, and humans with an unusual or attractive appearance. Each expert identified unacceptable scenes, and 35 scenes were removed from the sample pool. An investigator used a Microsoft Excel program to randomly select five photographs from the remaining scenes for this research (Figures 2, 3).

### Environmental Interventions

Using the five photographs of existing urban alleys, we developed 15 environmental interventions in three categories: Cleaning, Vegetation, and Urban Function. The Cleaning and Vegetation interventions, which improve the appearance of the place, are grounded in the Broken Windows Theory. The Urban Function interventions are grounded in the Routine Activities Theory because they focus on changing the function and facilitating routine activities in the place.

**Figure 4:**
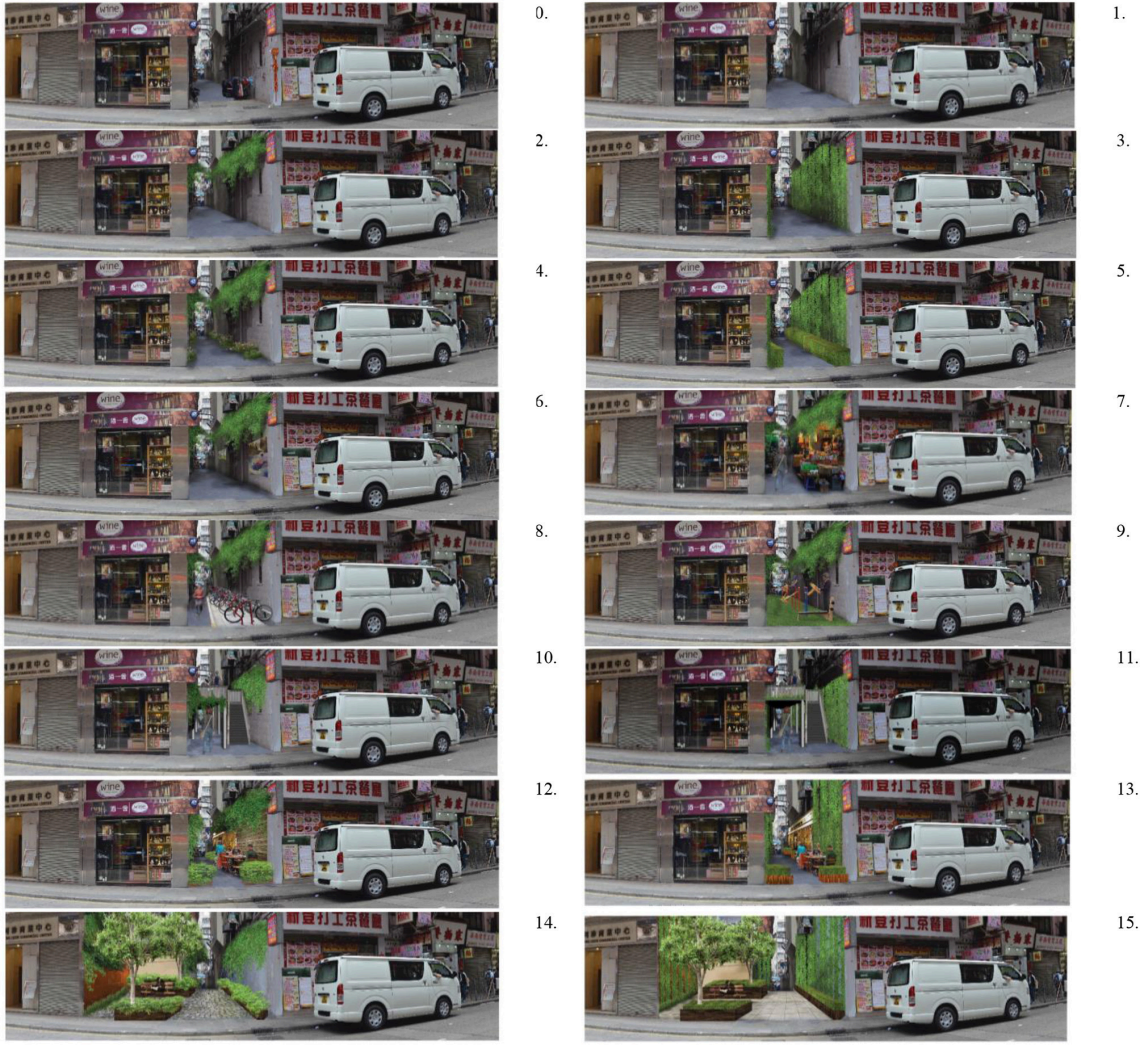
The baseline image and 15 simulated images for one site. 0, baseline; 1, cleaning; 2, naturalistic green wall; 3, geometric green wall; 4, naturalistic green wall & ground; 5, geometric green wall & ground; 6, green art gallery; 7, green shop; 8, green cycling; 9, green gym; 10, naturalistic green platform; 11, geometric green platform; 12, naturalistic green café; 13, geometric green café; 14, naturalistic green park; 15, geometric green park.

First, we assessed the impact of cleaning on perceived safety by comparing the current disorderly scene (“Baseline” scene, Figure 4, 0) with a cleaned scene (“Cleaning” intervention, Figure 4, 1). The “Cleaning” scene shows urban alleys after litter, dirt and graffiti are removed. This photograph was used as a background for the other 14 design interventions.

Second, we assessed the impact on perceived safety of adding various doses and styles of vegetation to the cleaned urban alley scenes. The vegetation interventions (“Vegetation,” Figure 4, 2–5) include four scenes: “naturalistic green wall,” “geometric green wall,” “naturalistic green wall and ground,” and “geometric green wall and ground.” We selected different kinds of vegetation (orderly, geometric and natural) because the orderliness of the vegetation may impact safety perceptions.

Third, we assessed the impact of urban functions in urban alley interventions on perceived safety. Three types of common urban functions were considered: small businesses, community facilities, and public spaces created by the government. The first two functions are bottom-up interventions by apartment owners, commercial tenants, or community organizations. Based on our observations of common small businesses in Hong Kong, many small business might spatially and functionally fit in the narrow urban alleys and media reported those functions can occasionally be found in existing urban alleys in Hong Kong (Creer, 2015), including cafes, newspaper stalls, coffee shops, bookstores, grocery stores, and snack shops. Community facilities may include outdoor exercise spaces, bike parking, and community art galleries.

Turning urban alleys into public spaces can be challenging in high-density cities like Hong Kong. Changes in building structure and street layout can be extremely difficult and expensive, requiring heavy investment and negotiations among stakeholders and the government. City planners have a few practical, less costly intervention options, and we propose two of them. First, they can transform the entry area of a urban alley into a small pocket park. Second, they can develop public space vertically by creating a linear platform over the urban alley. This type of intervention is observed in contemporary public space projects, but its impact on safety perception is unknown (“Designboom Architecture,” 2011).

We propose ten urban function interventions: shops, bike parking and paths, outdoor exercise facilities, outdoor cafes, outdoor art galleries, pocket parks, and public platforms over the urban alley (“Urban Function and Landscape” intervention scenes, Figure 4, 5–15).

### Photo Simulations

We used Adobe Photoshop 6.0 to create a panoramic photo of the existing scene for each site. Using these panoramic photos, we created photomontages, computerized visual simulations of urban alley revitalization scenarios (Figure 4). Human figures were included in each of the simulations to show how a space might be used. We used four criteria in producing the photo simulations: first, we chose common plant species without strong cultural meaning; second, we chose human figures with common hairstyles and clothes and avoided using human figures with an attractive or striking appearance; third, we chose ordinary materials for the simulation, not ones with an ascetic or luxurious appearance. For each of the five urban alleys, we simulated 15 types of revitalization scenarios in Adobe Photoshop CS 6. In total, 80 panoramic photos were used in the photo questionnaire.

### Photo-Questionnaire Survey

To recruit participants for the photo-questionnaire survey, each participant was randomly assigned to view 30 of the 80 photos in random order. Then the respondent was asked to rate their perceived safety for each scene on a five-point Likert scale ranging from “very unsafe (1)” to “very safe (5).” The safety rating is regarded as a positive perception if the score is greater than 3, a negative perception if the score is less than 3, and a neutral perception if the score equals 3. The 5-point Likert Scale has been repeatedly proven as a friendly scale for ordinary participants. Compared to a Likert scale with more points, it increases the response rate and quality and minimizes respondent frustration (Babakus and Mangold, 1992).

### Survey Respondents

We recruited 218 adults for the questionnaire survey through emails and flyers. Table 1 shows the socio-demographic characteristics of participants. Most of them are Hong Kong residents at young or middle age.

**Table 1:** Sample socio-demographic characteristics for participants.

**Demographic variables**	**Number**	**Percentage**
Sample size	218	100
**AGE**		
18–20	6	2.8
21–25	97	44.5
26–30	41	18.8
31–35	18	8.3
36–40	12	5.5
41–45	4	1.8
46–50	10	4.6
51–55	13	6.0
56–60	3	1.4
61–65	5	2.3
66–70	3	1.4
71 or above	6	2.8
**GENDER**		
Female	124	56.9
Male	94	43.1
**ETHNICITY**		
Chinese (Hong Kong SAR, China)	192	88.1
Chinese (Mainland China)	21	9.6
Persian	2	0.9
Filipino, Nepalese, Indian, or Indonesian	2	0.9
Black	1	0.5
White	0	0
Others	0	0
**MARITAL STATUS**		
Married	53	24.3
Window	2	0.9
Divorced	2	0.9
Single	158	72.5
**CRIME OR VIOLENT EXPERIENCE HAPPENED TO RESPONDENT OR**		
**RESPONDENT'S FAMILY OR FRIENDS IN THE LAST 2 YEARS**		
Yes	10	4.6
No	201	92.2
Missing data	7	3.2
**TO WHAT EXTENT DOES THE RESPONDENT FEEL FAMILIAR WITH THE**		
**URBAN ALLEY SCENES SHOWN IN THE QUESTIONNAIRE?**		
Very unfamiliar	23	10.6
Unfamiliar	75	34.4
Not sure	22	10.1
Familiar	85	39.0
Very familiar	13	6.0
**ARE THERE SIMILAR URBAN ALLEYS IN THE RESPONDENT'S DAILY**		
**ENVIRONMENT?**		
Almost not	12	5.5
A few	53	24.3
Some	79	36.2
Many	60	27.5
A lot	14	6.4

*A row named as “missing data” is presented if there are missing data for a question*.

## Results

Results are presented in five parts. First, we present the descriptive data and then compare the overall safety perception ratings between the 15 intervention scenes and the baseline scene. Second, we present the effect of the Cleaning intervention on safety perceptions. Third, we report the effect of the Vegetation interventions. Fourth, we present the effect of the Urban Function and Vegetation interventions. The comparison of intervention effects was realized through the Summary *T*-Test in IBM SPSS 23.

### Difference Between the 15 Interventions and the Baseline Scene

**Figure 5:**
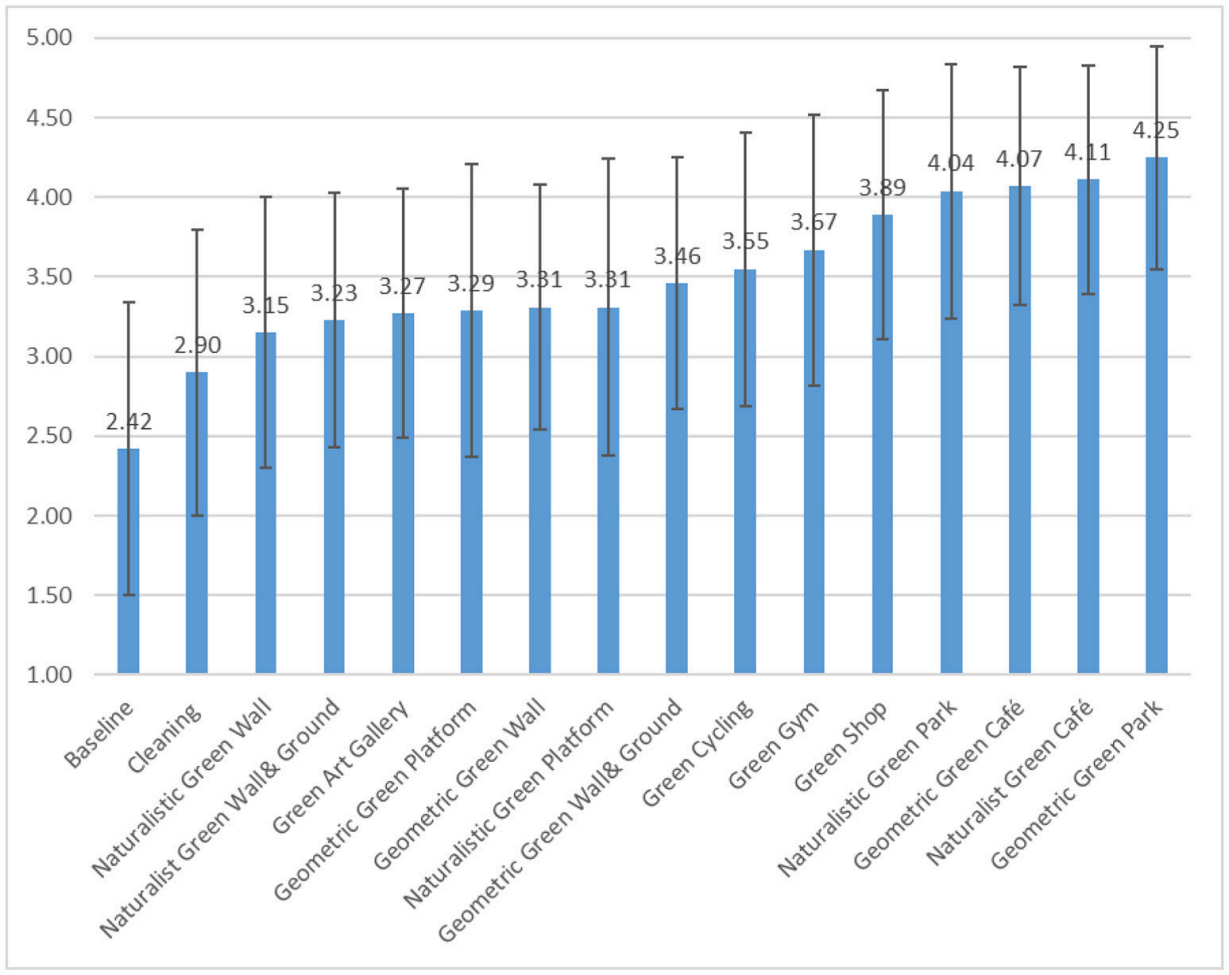
Average perceived safety of the baseline and intervention scenes ranging from low to high scores. Compared to the baseline, all interventions yielded significant promotion on perceived safety but effect size varies. Error bars represent ±1 standard deviation.

Compared to the baseline scene (current conditions of the urban alley), did the intervention scenes increase people's perceived safety? Table 2 and Figure 5 show the average perceived safety of the baseline scene and the 15 intervention scenes. In general, all interventions yield a significant increase in reported perceived safety, compared to the baseline (*p* < 0.0001).

**Table 2:** Descriptive information of the reported perceived safety for the baseline and 15 interventions and percent increase in perceived safety.

**Categories**	**Scenes**	***N***	**Avg. Mean**	**Mean**	**S.D**.	**S.E**.	**% Increase in safety**	**Ranking of safety**
Baseline	Baseline	307	2.42	2.42	0.92	0.05		16
Cleaning	Cleaning	295	2.90	2.90	0.90	0.05	20^***^	15
Vegetation	Naturalistic Green Wall	340	3.29	3.15	0.85	0.05	30^***^	14
	Geometric Green Wall	293		3.31	0.77	0.04	37^***^	9
	Naturalist Green Wall and Ground	348		3.23	0.80	0.04	34^***^	13
	Geometric Green Wall and Ground	335		3.46	0.79	0.04	43^***^	8
Urban function	Green Art Gallery	332	3.75	3.27	0.78	0.04	35^***^	12
	Green Shop	345		3.89	0.78	0.04	61^***^	5
	Green Cycling	327		3.55	0.86	0.05	47^***^	7
	Green Gym	349		3.67	0.85	0.05	52^***^	6
	Naturalistic Green Platform	324		3.31	0.93	0.05	37^***^	10
	Geometric Green Platform	309		3.29	0.92	0.05	36^***^	11
	Naturalist Green Café	335		4.11	0.72	0.04	70^***^	2
	Geometric Green Café	336		4.07	0.75	0.04	68^***^	4
	Naturalistic Green Park	332		4.04	0.80	0.04	67^***^	3
	Geometric Green Park	331		4.25	0.70	0.04	76^***^	1

Percent increase in safety = perceived safety of an intervention scene/perceived safety of the baseline scene × 100%.

*** *<0.001*.

### The Validity of the Four Categories

**Figure 6:**
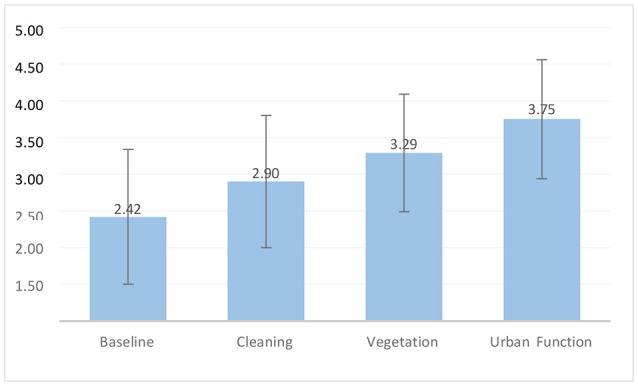
Average perceived safety of the baseline and three categories of interventions ranging from low to high scores. A rating greater than 3.00 is a positive perception and lower than 3.00 is a negative perception. Error bars represent ±1 standard deviation.

Figure 6 shows a clear trend of increasing perceived safety across the four categories: Baseline (*M* = 2.42, *SD* = 0.92), Cleaning (*M* = 2.90, *SD* = 0.90), Vegetation (*M* = 3.29, *SD* = 0.80), and Urban Function (*M* = 3.75, *SD* = 0.81). We wanted to see if each group was significantly different in participants' rating scores from the other groups. We conducted an *F*-test within the four categories for all participants and male and female samples (12 tests in total). All 12 tests resulted in *p-*values of < 0.01 or < 0.001. This suggests that the ratings scores are significantly different for each of the categories (Table 3), and that our categorization is reliable.

**Table 3:** *F*-test between each pair of four categories of interventions.

**All**	**df**	**rss_0**	**rss_a**	***F***
1v2	1, 600	509.91	498.80	13.37^***^
1v3	4,1,618	1369.64	1111.01	94.16^***^
1v4	10, 3,616	3354.71	2444.83	134.58^***^
2v3	4, 1,606	1101.15	1090.11	4.06^**^
2v4	10, 3,604	2945.09	2423.94	77.49^***^
3v4	13, 4,622	3682.26	3036.15	75.66^***^

1, Baseline, 2, Cleaning, 3, Vegetation, 4, Urban Function.

^*^< 0.05,

** < 0.01,

*** *< 0.001*.

### Effect of Cleaning Intervention and Vegetation Interventions

The Cleaning intervention yields a positive increase in perceived safety, compared to the baseline scene, *t*_(1, 600)_ = −6.47, *p* < 0.001. However, the perceived safety value is 2.90, which is lower than the neutral value (3.00), suggesting that participants still perceive the Cleaning scene as somewhat unsafe.

Compared to the Cleaning scene, adding either geometric or naturalistic plants on the urban alley walls yields a significant increase in perceived safety. The effect of naturalistic plants on the ground is *t*_(1, 633)_ = −3.60, *p* < 0.001, an increase of 9% from the Cleaning scene. The effect of the geometric plants on the ground is *t*_(1, 586)_ = 5.93, *p* < 0.001, a 14% increase from the Cleaning scene. However, the four Vegetation interventions rank 7th, 8th, 12th, and 13th among all 15 interventions, and safety scores range from 3.15 to 3.46, suggesting their effects are moderate.

Adding additional ground-level plants showed a mixed effect on further promotion of perceived safety. Adding naturalistic ground-level plants to the naturalistic green wall scene yields an insignificant effect, *t*_(1, 686)_ = 1.27, *p* = 0.20. Adding ground-level geometric plants to the geometric green wall scene yields a 5% increase in perceived safety, and the effect is significant, *t*_(1, 626)_ = 2.43, *p* < 0.05.

### Effects of Urban Function Interventions

The Vegetation interventions do yield greater increases in safety promotion, but will vegetation interventions that are paired with urban functions yield even greater increases in perceived safety? As shown in Table 4, all urban function interventions paired with vegetation interventions promote greater perceived safety than the Cleaning intervention. We choose to compare all urban function interventions with the vegetation intervention that yielded the highest score on perceived safety: Geometric Green Wall and Ground. Moreover, 6 out of 10 urban interventions yield significantly greater safety promotion effects than the Geometric Green Wall and Ground: The Green Shop intervention, the two Green Parks, the two Green Café interventions, and the Green Outdoor Gym. One urban function intervention yields a greater but non-significant promotion effect (the Green Cycling intervention). The other three urban interventions yield a slightly lower safety promotion effect.

**Table 4:** Comparing Urban Function interventions with the most effective Vegetation intervention (Geometric Green Wall and Ground).

**Scenes**	***N***	**Mean**	**S.D**.	**df**	***t***	**Increased safety (%)**
Geometric Green Wall and Ground	335	3.46	0.79		
Green Art Gallery	332	3.27	0.78	665	−3.13^**^	−5
Green Shop	345	3.89	0.78	678	7.14^***^	12
Green Cycling	327	3.55	0.86	660	1.40^ns^	3
Green Outdoor Gym	349	3.67	0.85	682	3.34^***^	6
Naturalistic Green Platform	324	3.31	0.93	657	−2.23^*^	−4
Geometric Green Platform	309	3.29	0.92	642	−2.52^*^	−4
Naturalistic Green Café	335	4.11	0.72	668	11.13^***^	19
Geometric Green Café	336	4.07	0.75	669	10.26^***^	18
Naturalistic Green Park	332	4.04	0.80	665	−9.42^***^	17
Geometric Green Park	331	4.25	0.70	664	13.65^***^	23

Increased safety = perceived safety of an Urban Function intervention scene/ Geometric Green Wall and Ground scene × 100%. ns, non-significant,

* < 0.05,

** < 0.01,

*** *< 0.001*.

### Different Effects of Naturalistic and Geometric Scenes

We further compared whether the two geometric and two naturalistic vegetation intervention scenes are significantly different in promoting perceived safety. For the wall vegetation interventions, geometric vegetation yields a 5% greater increase in perceived safety than the naturalistic vegetation, *t*_(1, 631)_ = 2.47, and *p* < 0.05. For the vertical and ground-level vegetation interventions, geometric vegetation yields a 7% increase in perceived safety than naturalistic vegetation, *t*_(1, 626)_ = 2.40, and *p* < 0.001.

We also compared the geometric and naturalistic Urban Function interventions. For the community park interventions, a geometric style yields a 5% greater promotion effect than the naturalistic style, *t*_(1, 661)_ = 3.60, and *p* < 0.001. For the cafe interventions, there is no significant difference between the geometric and naturalistic vegetation, *t*_(1, 669)_ = −0.71, *p* = 0.48. For the platform interventions, there is also no significant difference, *t*_(1, 631)_ = −0.27, *p* = 0.79.

### Statistical Clustering Analysis

Although our categorization of the scenes into four categories (baseline, Cleaning, Vegetation, and Urban Function) is statistically reliable in predicting the overall differences in safety perception, we found some mixture effects among several Vegetation and Urban Function interventions. That is, some of the Vegetation and Urban Function scores were very similar. We wondered if a different categorization could better predict the differences in safety perception and provide urban managers and designers with more specific suggestions for choosing the most effective intervention(s). We put 16 scenes from the Vegetation and Urban Function interventions in a more finely-sorted hierarchy using a k-means clustering method (Nowakowski et al., 2014). The prototype of the clustering analysis is to partition n observations into k clusters, where each observation belongs to the cluster with the nearest mean.

**Figure 7:**
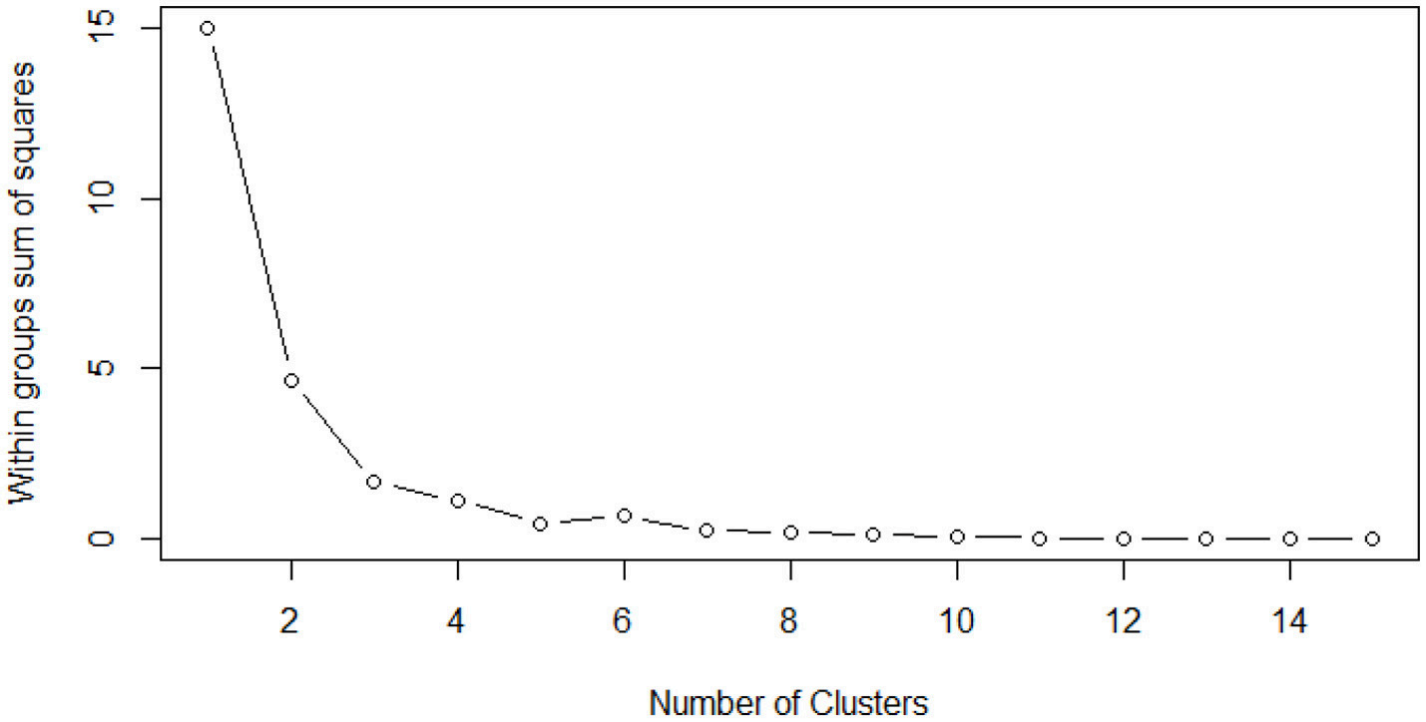
The within groups sum of square reaches a stable low level as the number of clusters reaches 5, which indicates 5 is an optimal amount for k-means clusters.

To see how many clusters are appropriate, we first conducted a within-group sum of squares test for different group sizes. The results in Figure 7 show that the clustering quality indicated by the within-group sum of square stays consistent after 5 or more groups. Thus, we decided to group the data into 5-group clusters. For comparison, we also conducted 4-group clustering, the number of our original categorization.

Compared to 4-group clustering, 5-group clustering shows a better performance, indicated by a higher *F*-value and a clearer separation between Vegetation and Urban Function and Vegetation interventions (Table 5). In general, either 4-group or 5-group clustering shows a similar clustering as the original 4 groupings, but we found similar effects between the higher rated Vegetation interventions (the two geometric vegetation interventions) and the lower rated Urban Function and Vegetation interventions (the art gallery, cycling, and platform interventions), warranting a separate category.

**Table 5:** Statistical clustering of 16 scenes: results of the four and five K-means cluster analysis.

**Clusters**	**Four clusters**		**Five clusters**	
	**Sub-groups**	**Mean**	**Sub-groups**	**Mean**
1st	Baseline	2.42	Baseline	2.42
2nd	Cleaning Naturalistic Green Wall	3.03	Cleaning	2.90
3rd	Naturalist Green Wall & Ground Green Art Gallery Geometric Green Platform Geometric Green Wall Naturalistic Green Platform Geometric Green Wall & Ground Green Cycling Green Gym	3.39	Naturalist Green Wall & Ground Green Art Gallery Geometric Green Platform Geometric Green Wall Naturalistic Green Platform	3.26
4th	Green Shop Naturalist Green Café Geometric Green Café Naturalistic Green Park Geometric Green Park	4.07	Geometric Green Wall & Ground Green Cycling Green Gym	3.56
5th	N/A		Green Shop Naturalist Green Café Geometric Green Café Naturalistic Green Park Geometric Green Park	4.07
Summary	*F*_(3, 12)_ = 49.71, *p* < 0.001		*F*_(4, 11)_ = 89.19, *p* < 0.001	

## Discussion

This study identifies the impact of three types of urban alley interventions, based on the Broken Windows Theory and Routine Activity Theory, on citizens' perceived safety. In the following sections, we discuss the impacts of the three types of interventions on perceived safety and present possible explanations. Then we discuss the main implications of this study. Lastly, we suggest opportunities for future research.

### Theoretical Interpretation of Impacts of Interventions

#### Cleaning Intervention

The promotion effect of the Cleaning intervention is statistically significant but has a small effect size, which suggests people still do not perceive the urban alley as safe, even after cleaning. This finding is only partially consistent with BWT which suggests that cleanliness will improve the perceived safety of a space by delivering the social message that the place is cared for, which will discourage crime (Wilson and Kelling, 1982). The findings clearly suggest this intervention based on BWT has a positive impact on perceived safety but the impact is not powerful enough.

#### Vegetation Interventions

The four Vegetation interventions promote perceived safety even more. Adding vegetation to urban alleys can promote perceived safety, which is consistent with the Broken Windows Theory. According to this theory, vegetation sends a message that the place is well cared for and more inviting for legitimate users. This result also agrees with previous studies suggesting that greening can remediate spaces and enhance perceived safety in urban alleys (Seymour et al., 2010; Wolch et al., 2010; Wolfe and Jeremy, 2012) and other urban settings, including urban public housing, vacant street lots, and parks (Kuo et al., 1998; Yang et al., 2013).

However, the promotion effects of vegetation are only moderate. One possible reason is that the Vegetation interventions only change the visual appearance of the urban alleys, but do not transform the ambiguous and enigmatic spaces into ones with a clear identity and routine use. This explanation is supported by empirical studies in urban environmental design that argue that ambiguity and ignorance are two main challenges for safety in urban urban alleys (Seymour et al., 2010; Wolch et al., 2010; Cozens and Love, 2015). Once again, interventions based on BWT positively impact perceived safety but the impact is not powerful enough.

#### Urban Function Interventions

The addition of urban functions (shops, cycling, gyms, cafés, art galleries, parks, or platforms), interventions grounded in RAT, yields a further increase in perceived safety. This finding is consistent with a study that found perceived safety is positively associated with the amount of public facilities and related organized activities (Sandra et al., 2015). Our finding also echoes findings of an onsite, experimental study that adding functional public spaces in streets can promote residents' perceived safety and reduce actual crime rates (Branas et al., 2011, 2018). In particular, we found the urban function interventions that accommodate more legitimate users and a longer duration of supervision were perceived as safer.

This finding can be explained in two ways. First, the Urban Function interventions provide inviting public spaces that attract residents and visitors. The activities that flourish in such places would make the space more interactive, invite more vigorous formal supervision, and discourage social incivilities, thereby promoting perceived safety (Brunson et al., 2001; Sreetheran and van den Bosch, 2014). Second, active public spaces often facilitate a stronger sense of stewardship, which can encourage environmental improvements. These improvements in turn stimulate a more active social life in the space. This cycle can enhance perceived safety in a sustainable way (Francis et al., 2012; Jorgensen et al., 2013; Cozens and Love, 2015).

### A More Detailed Interpretation With Felson's Matrix

Felson's matrix of crime discouragement (1995), which compares the Broken Windows and Routine Activity theories, can provide a further explanation for the differences in perceived safety among the three categories of interventions.

According to the matrix, a substantial difference between interventions grounded in BWT and those grounded in RAT is the allocation of supervision responsibilities to inhibit deviance and crime. Felson (1995) suggests that safe spaces should include four levels of supervision responsibility in monitoring suitable targets, likely offenders, and amenable places: (1) personal (owners, family, or friends who have a personal investment in the places), (2) assigned (employees with specific assignments to monitor security), (3) diffuse (employees such as gardeners, waiters, or social workers with general assignments who may also keep an eye on security issues), and 4) general (strangers/other citizens who are visiting or walking through a space). Using this supervision responsibility matrix, we can further interpret differences in perceived safety for the Cleaning, Vegetation, and Urban Function interventions.

Compared to the baseline scene, the Cleaning intervention does not generate new personal and assigned supervision responsibilities, but it does add a low level of diffuse responsibility (someone may be assigned to briefly clean a site once or twice a day) and a low level of general responsibility (a few more passengers may walk through the urban alley, but they have no reason to stay). Thus, the Cleaning invention would promote greater supervision than the existing disorderly urban alleys, leading to greater perceived safety, but the amount of supervision would be quite limited.

Vegetation interventions would not likely generate more personal supervision responsibility because the vegetation in streets or urban alleys is usually paid for and installed by a government agency, not individuals or businesses (at least in Hong Kong). Security guards (assigned supervision responsibility) are not usually assigned when vegetation is added to a space. Vegetation may add a low level of diffuse supervision responsibility with the presence of part-time gardeners. The vegetation may also increase the presence of passers-by who seek contact with nature (general supervision responsibility), but the presence of visitors will depend on the aesthetic and restorative quality of the vegetation. A small amount of ordinary vegetation in a narrow urban alley may not be enough to transform an urban alley into an inviting space. Thus, compared to the Cleaning intervention, adding vegetation would further increase supervision and perceived safety, but its effect would also be limited.

In contrast, adding legitimate urban functions to the urban alleys would add personal supervision responsibility with the presence of business or property owners. The owner may hire at least one staff member to monitor the safety of his or her property and the surrounding vicinity. Other employees not assigned to security (e.g., shopkeepers, janitors) would have diffuse supervision responsibilities while working. Moreover, new functions or amenities would attract visitors or customers, increasing the number of eyes in the space (general supervision responsibility). It is important to note this type of intervention would not only increase the number of people with different supervision responsibilities but also increase the duration and frequency of supervision in the urban alleys. Thus, adding legitimate urban amenities or functions would increase the number of people with varying levels of supervision responsibility and thereby increase perceived safety.

### Interpretation on Difference Between Geometric and Naturalistic Scenes

Three out of five geometric scenes were perceived as significantly safer than the naturalistic scenes, including both Vegetation scenes and one of the three Urban Function scenes. These findings are consistent with previous studies showing preferences for geometric, orderly designs (Parsons, 1995; Mahidin and Maulan, 2012; Yang et al., 2013). One possible reason for this finding is that formal, manicured vegetation seems to indicate civilization, tidiness, clear intention, and maintenance (Shaffer and Anderson, 1985; Jansson et al., 2013). These attributes make people feel safer. The geometric, manicured vegetation may also imply surveillance by local residents, community and public sectors (more capable guardians), further promoting perceived safety (Yang et al., 2013). This explanation bridges the BWT and RAT, arguing that geometric, manicured vegetation not only signals orderliness but also the presence of human stewardship. However, it is important to note that landscapes with geometric or manicured vegetation might not be perceived as safer than naturalistic landscapes in other types of urban space. Preferences for naturalistic vs. geometric vegetation might be influenced by the specific functions and targeted users of various urban environments (Bixler and Myron, 1997; Gobster, 2001; Özgüner and Kendle, 2006).

### Key Contributions and Implications

In recent years, cities have increased urban alley revitalization projects. However, since these revitalization projects are relatively new, their effectiveness in promoting perceived safety has not been empirically tested. This study is an initial effort using criminal and environmental psychology literature to offer solid theoretical explanations for different levels of efficacy of interventions grounded in BWT or RAT.

In this study, we provide rationale to extend the content and implications of the Routine Activity Theory. RAT emphasizes the social structures that generate a convergence of three key factors (potential target, perpetrator, and guardians) in a comprehensive spatio-temporal model, but does not address the visual perception of scenes of a given site. We provide justification to extend the theory to explore people's safety perceptions of environmental interventions and emphasize that perceived safety can be improved by adding urban functions at the site scale.

RAT has rarely been used in environmental psychology research, where photographs or rendered images are used to represent social and ecological characteristics of a site (Ulrich, 1981; Hartig et al., 1997; van den Berg and Vlek, 1998; Sullivan and Lovell, 2006; Korpela, 2013; Jiang et al., 2015). We argue that photographs as surrogates of actual settings provide hints of the social structure of the place. Humans have the ability to infer the social structure from the content and organization of visual elements (Kaplan et al., 1998). For example, by looking at the photographs, people can speculate the number of service hours, business owners and employees, and presence of legitimate citizens, although they may not be visually present in the photograph. Based on these inferences, people can estimate safety risks.

Finally, we present strong evidence that interventions based on the BWT are limited in their ability to promote perceived safety. Compared to urban function interventions based on RAT, removal of disorder has a much smaller effect. This idea challenges conventional wisdom that simply cleaning and greening urban alleys (and other places) is enough. To improve perceived safety, we must also provide clear and diverse urban functions.

### Opportunities for Future Research

We used simulations from photographs of existing urban alley conditions as the main tool for measuring perceived safety. Some scholars argue that viewing a photograph does not fully capture the multi-sensory experience of being on site. Also, the static photograph cannot fully convey dynamic information of a site, information that may affect people's perceived safety (Sreetheran and van den Bosch, 2014). However, other studies suggest viewing photographs can be a reliable surrogate for on-site experience (Kuo et al., 1998; Hernández and Hidalgo, 2005; Nielsen et al., 2012; Jiang et al., 2016). Future research should use more immersive and interactive surrogates, such as 3D imagery (Jiang et al., 2014) or interactive virtual reality technology (Valtchanov et al., 2010).

In our study, urban function interventions included vegetation. Do interventions that contain urban functions but little or no vegetation still promote perceived safety? Future research should test this possibility. In addition, future studies should further investigate the impact of the density of geometric and naturalistic vegetation on perceived safety.

The Vegetation interventions did not include more active and engaging landscapes, such as productive or therapeutic gardens. Future research should evaluate these “active” vegetation interventions because they may increase eyes on the street and informal guardianship. Also, researchers should investigate how time influences safety perceptions. Previous studies note that people might have different perceptions of safety in the day and the night (Brands et al., 2013; Sreetheran and van den Bosch, 2014). The impacts of night scenes with different lighting conditions should also be examined (Nasar and Bokharaei, 2017).

Ownership of the urban alleys is complicated and inevitably challenges environmental intervention projects. Future research should look into land ownership issues and examine the practicality of urban alley revitalization projects. Also, future research should consider developing specific policies to promote small business in urban alley environments.

The design of the questionnaire may have created some bias, because respondents evaluated similar alley scenes with similar types of interventions for 30 photographs and answered the same question for each photograph. Although respondents were randomly assigned to view 30 of the 80 photographs, this does not entirely remove bias. Future research should address this limitation through a more carefully designed questionnaire.

The alleys we photographed are typical of alleys in modern high-density cities. We excluded alleys with significant cultural or geographic specificity in this study. The interventions we selected are common and are found in many urban revitalization projects in different regions (Newell et al., 2013). Therefore, we have confidence in our study's generalizability. However, it is necessary for future researchers to replicate this study in other cities to fully ensure wide applicability. Finally, we examined perceived safety, not actual safety in this study. We suggest that future researchers investigate how the environmental interventions we explore impact actual deviant behavior and crime.

## Conclusion

This study provides both a theoretical foundation and empirical evidence to guide the design of urban alleys. We quantify the impacts of a wide variety of urban alley design interventions on perceived safety, using a theoretical framework to propose design interventions and interpret perceptions. We argue that cleaning or adding plants in urban alleys is helpful, but changing the visual appearance of the space is far from enough. It is critical that spaces also generate active, diverse, and prolonged legitimate routine activities, adding multiple layers of formal and informal surveillance to the space. We argue that interventions based on the Broken Windows Theory have limited efficacy in improving perceived safety, and policy-makers and environmental designers should adopt urban function interventions motivated by the Routine Activity Theory to create safer urban spaces.

## Ethics Statement

The study was approved by Human Research Ethics Committee at the University of Hong Kong. Participants were residents in Hong Kong. First, potential participants were shown an introduction of the main purpose, meaning, procedure, potential risk, investigators, and contact information. The introduction was written in both English and Chinese. Second, they were asked by an investigator to read the material carefully. Last, they chose to answer the questionnaire only if they agree to participate. They had the right to stop their participation at any time.

## Author Contributions

BJ and CM initiated the research idea and developed the research methods together. CM conducted the site investigation and data collection. CM and BJ run the first round of data analysis and then BJ run the second and third round of data analysis. CM wrote the first draft and BJ led the writing of the second, third, and fourth round of manuscript with support from HZ, LL, and CW. HZ, CW, and LL also provided important suggestions on presentation of the theoretical framework, data analysis, and interpretation of findings.

### Conflict of Interest Statement

The authors declare that the research was conducted in the absence of any commercial or financial relationships that could be construed as a potential conflict of interest.

## Footnotes

^1^The progressive sub-division of densely populated urban spaces over the centuries has created pedestrian-scale thoroughfares between lots and buildings. Access in newer high density subdivisions has tended to be achieved through rear and side alleys (referred to as “back alleys” in some regions). In the northern industrial cities of England, alleys are called “tenfooters” because that was the dimension thought safe for fire, services and public health purposes. We use the widely-used term, “urban alley” (Seymour et al., 2010; Newell et al., 2013).

## References

[B1] Babakus E. Mangold W. G. (1992). Adapting the SERVQUAL Scale to Hospital Services: an empirical investigation. Health Serv. Res. 26,767–780. 1737708 PMC1069855

[B2] Bixler R. D. Myron F. F. (1997). Nature is scary, disgusting, and uncomfortable. Environ. Behav. 29, 443–467. 10.1177/001391659702900401

[B3] Branas C. C. Cheney R. A. MacDonald J. M. Tam V. W. Jackson T. D. Ten Have T. R. (2011). A difference-in-differences analysis of health, safety, and greening vacant urban space. Am. J. Epidemiol. 174, 1296–1306. 10.1093/aje/kwr273 22079788 PMC3224254

[B4] Branas C. C. South E. Kondo M. C. Hohl B. C. Bourgois P. Wiebe D. J. (2018). Citywide cluster randomized trial to restore blighted vacant land and its effects on violence, crime, and fear. *Proc. Natl. Acad. Sci*. U.S.A. 115, 2946–2951. 10.1073/pnas.1718503115 PMC5866574 29483246

[B5] Brands J. Schwanen T. van Aalst I. (2013). Fear of crime and affective ambiguities in the night-time economy. Urban Stud. 52, 439–455. 10.1177/0042098013505652

[B6] Brunson L. Kuo F. E. Sullivan W. C. (2001). Resident appropriation of defensible space in public housing: implications for safety and community. Environ. Behav. 33, 626–652. 10.1177/00139160121973160

[B7] Cohen L. E. Felson M. (1979). Social change and crime rate trends: a routine activities approach. Am. Sociol. Rev. 44, 588–608. 10.2307/2094589

[B8] Coley R. L. Sullivan W. C. Kuo F. E. (1997). Where does community grow? the social context created by nature in urban public housing. Environ. Behav. 29, 468–494. 10.1177/001391659702900402

[B9] Cozens P. Love T. (2015). A review and current status of crime prevention through environmental design (CPTED). J. Plan. Lit. 30, 393–412. 10.1177/0885412215595440

[B10] Creer D. (2015). Hong Kong Alleys. Available online at: http://www.danielgreer.photography/hong-kong-alleys/ (Accessed 8, October 2016)

[B11] Daley R. M. (2007). Chicago Green Alley Handbook: An Action Guide to Create a Greener, Environmentally Sustainable. Chicago, IL: Chicago Department of Transportation.

[B12] Donovan G. H. Prestemon J. P. (2012). The effect of trees on crime in Portland, Oregon. Environ. Behav. 44, 3–30. 10.1177/0013916510383238

[B13] Doran B. J. Burgess M. B. (2012). Putting Fear of Crime on the Map. London: Springer.

[B14] Felson M. (1995). Those who discourage crime. Crime Place 4, 53–66.

[B15] Foster S. Giles-Corti B. Knuiman M. (2014). Does fear of crime discourage walkers? A social-ecological exploration of fear as a deterrent to walking. Environ. Behav. 46, 698–717. 10.1177/0013916512465176

[B16] Francis J. Giles-Corti B. Wood L. Knuiman M. (2012). Creating sense of community: the role of public Space. J. Environ. Psychol. 32, 401–409. 10.1016/j.jenvp.2012.07.002

[B17] Furr-Holden C. D. Lee M. H. Milam A. J. Johnson R. M. Lee K. S. Ialongo N. S. (2011). The growth of neighborhood disorder and marijuana use among urban adolescents: a case for policy and environmental interventions. J. Stud. Alcohol Drugs 72, 371–379. 10.15288/jsad.2011.72.371 21513673 PMC3084354

[B18] Gau J. M. Pratt T. C. (2010). The growth of neighborhood disorder and marijuana use among urban adolescents: a case for policy and environmental interventions. J. Crim. Justice 38 , 758–766 10.1016/j.jcrimjus.2010.05.002 PMC3084354 21513673

[B19] Gobster P. H. (2001). Visions of nature: conflict and compatibility in urban park restoration. Landsc. Urban Plan. 56, 35–51. 10.1016/S0169-2046(01)00164-5

[B20] Hartig T. Korpela K. Evans G. W. Garling T. (1997). A measure of restorative quality in environments. Scand. Housing Plan. Res. 14, 175–194. 10.1080/02815739708730435

[B21] Hernández B. Hidalgo M. C. (2005). Effect of urban vegetation on psychological restorativeness. Psychol. Rep. 96, 1025–1028. 10.2466/pr0.96.3c.1025-1028 16173374

[B22] Hinkle J. C. Yang S. M. (2014). A new look into broken windows: what shapes individuals' perceptions of social disorder? J. Crim. Justice 42, 26–35. 10.1016/j.jcrimjus.2013.11.002

[B23] Hong Kong Police Force (2014). Hong Kong Police Review, 2014, Available online at: http://www.police.gov.hk/info/review/2014/tc/index.html#111/z

[B24] Jacobs J. (1961). The Death and Life of Great American Cities. New York, NY: Random House.

[B25] Jansson M. Fors H. Lindgren T. Wiström B. (2013). Perceived personal safety in relation to urban woodland vegetation–a review. Urban Forestry Urban Green 12, 127–133. 10.1016/j.ufug.2013.01.005

[B26] Jiang B. Chang C.-Y. Sullivan W. C. (2014). A dose of nature: tree cover, stress reduction, and gender differences. Landsc. Urban Plan. 132, 26–36. 10.1016/j.landurbplan.2014.08.005

[B27] Jiang B. Larsen L. Deal B. Sullivan W. C. (2015). A dose–response curve describing the relationship between tree cover density and landscape preference. Landsc. Urban Plan. 139, 16–25. 10.1016/j.landurbplan.2015.02.018

[B28] Jiang B. Li D. Larsen L. Sullivan W. C. (2016). A dose-response curve describing the relationship between urban tree cover density and self-reported stress recovery. Environ. Behav. 48, 607–629. 10.1177/0013916514552321

[B29] Jorgensen L. J. Ellis G. D. Ruddell E. (2013). Fear perceptions in public parks. Environ. Behav. 45, 803–820. 10.1177/0013916512446334

[B30] Kaplan R. Kaplan S. Ryan R. (1998). With People in Mind- Design and Management of Everyday Nature. Washington, DC; Covelo, CA: Island Press.

[B31] Keizer K. Lindenberg S. Steg L. (2008). The spreading of disorder. Science 322, 1681–1685. 10.1126/science.1161405 19023045

[B32] Korpela K. M. (2013). Perceived restorativeness of urban and natural scenes - Photographic illustrations. J. Archit. Plann. Res. 30, 23–38.

[B33] Kotabe H. P. (2014). The world is random: a cognitive perspective on perceived disorder. Front. Psychol. 5:606. 10.3389/fpsyg.2014.00606 24982648 PMC4056180

[B34] Kuo F. E. Bacaicoa M. Sullivan W. C. (1998). Transforming inner-city vegetation trees, perceived safety, and preference. Environ. Behav. 30, 28–59. 10.1177/0013916598301002

[B35] Kuo F. E. Sullivan W. C. (2001). Environment and crime in the inner city does vegetation reduce crime? Environ. Behav. 33, 343–367. 10.1177/00139160121973025

[B36] Madge C. (1997). Public parks and the geography of fear. Tijdschrift voor economische sociale geografie 88, 237–250. 10.1111/j.1467-9663.1997.tb01601.x

[B37] Mahidin A. M. M. Maulan A. (2012). Understanding children preferences of natural environment as a start for environmental sustainability. Procedia-Soc. Behav. Sci. 38, 324–333. 10.1016/j.sbspro.2012.03.354

[B38] Meghan E. H. Marcus F. Brandon C. W. (2013). The capable guardian in routine activities theory: a theoretical and conceptual reappraisal. Crime Prev. Community Safety 15:65 10.1057/cpcs.2012.14

[B39] Mok D. (2012). Manhunt after Girl Is Raped in Wan Chai. South China Morning Post. October, 2015. Available online at: http://www.scmp.com/article/991357/manhunt-after-girl-raped-wan-chai.

[B40] Nasar J. L. Bokharaei S. (2017). Impressions of lighting in public squares after dark. Environ. Behav. 49, 227–254. 10.1177/0013916515626546

[B41] Nasar J. L. Fisher B. (1993). ‘Hot spots' of fear and crime: a multi-method investigation. J. Environ. Psychol. 13, 187–206.

[B42] Nathanson B. Emmet D. (2007). Alley Gating and Greening Toolkit Baltimore. Ashoka: Innovators for the Public.

[B43] Newell J. P. Seymour M. Yee T. Renteria J. Longcore T. Wolch J. R. (2013). Green urban alley programs: planning for a sustainable urban infrastructure? Cities 31, 144–155. 10.1016/j.cities.2012.07.004

[B44] Nielsen A. B. Heyman E. Richnau G. (2012). Liked, disliked and unseen forest attributes: relation to modes of viewing and cognitive constructs. J. Environ. Manage. 113,456–466. 10.1016/j.jenvman.2012.10.014 23122619

[B45] Nowakowski T. Młyńczak M. Jodejko-Pietruczuk A. Werbińska-Wojciechowska S. (eds.). (2014). Safety and Reliability: Methodology and Applications. CRC Press.

[B46] O'Brien D. T. (2015). Disorder perception is the adaptive interpretation of social cues, not just a sensitivity to randomness. Front. Psychol. 6:124 10.3389/fpsyg.2015.00124 25713556 PMC4322537

[B47] O'Brien D. T. Norton C. C. Cohen J. Wilson D. S. (2014). Local adaptation in community perception: how background impacts judgments of neighborhood safety. Environ. Behav. 46, 213–240. 10.1177/0013916512456844

[B48] O'Brien D. T. Sampson R. J. (2015). Public and private spheres of neighborhood disorder: assessing pathways to violence using large-scale digital records. J. Res. Crime Delinq. 52, 486–510. 10.1177/0022427815577835

[B49] O'Brien D. T. Wilson D. S. (2011). Community perception: the ability to assess the safety of unfamiliar neighborhoods and respond adaptively. J. Pers. Soc. Psychol. 100, 606–620. 10.1037/a0022803 21443374

[B50] Osgood D. W. Wilson J. K. O'Malley P. M. Bachman J. G. Johnston L. D. (1996). Routine activities and individual deviant behavior. Am. Sociol. Rev. 61, 635–655. 10.2307/2096397

[B51] Özgüner H. Kendle A. D. (2006). Public attitudes toward naturalistic versus designed vegetation in the city of Sheffield (UK). Landsc. Urban Plan. 74,139–157. 10.1016/j.landurbplan.2004.10.003

[B52] Parsons R. (1995). Conflict between ecological sustainability and environmental aesthetics: conundrum, canärd, or curiosity. Landsc. Urban Plan. 32, 227–244. 10.1016/0169-2046(95)07004-E

[B53] Pratt T. C. Turanovic J. J. (2016). Lifestyle and routine activity theories revisited: The importance of “Risk” to the study of victimization. Victims Offenders 11, 335–354. 10.1080/15564886.2015.1057351

[B54] Roncek D. W. Pamela A. M. (1991). Bars, blocks, and crimes revisited: linking the theory of routine activities to the empiricism of ‘hot spots'. Criminology 29, 725–753.

[B55] Rountree P. W. Kenneth C. L. (1996). Burglary victimization, perceptions of crime risk, and routine activities: a multilevel analysis across Seattle neighborhoods and census tracts. J. Res. Crime Delinq. 33, 147–180. 10.1177/0022427896033002001

[B56] Rucki A. (2014). Drugs, Cash and Weapons Discovered During Police Raid in East London Alley. Available online at: https://www.standard.co.uk/news/crime/drugs-cash-and-weapons-discovered-during-police-raid-in-east-london-alley-9764408.html (Accessed June 10, 2017).

[B57] Sampson R. J. Raudenbush S. W. (2004). Seeing disorder: Neighborhood stigma and the social construction of “broken windows”. Soc. Psychol. Q. 67, 319–342. 10.1177/019027250406700401

[B58] Sandra C. L. Deborah A. C. Bing H. (2015). How important is perception of safety to park use? A four-city survey. Urban Stud. 53, 2624–2636. 10.1177/0042098015592822 PMC8455087 34552299

[B59] Seymour M. Wolch J. Reynolds K. D. Bradbury H. (2010). Resident perceptions of urban alleys and urban alley greening. Appl. Geogr. 30, 380–393. 10.1016/j.apgeog.2009.11.002

[B60] Shaffer G. S. Anderson L. M. (1985). Perceptions of the security and attractiveness of Urban parking lots. J. Environ. Psychol. 5, 311–323. 10.1016/S0272-4944(85)80001-3

[B61] Skogan W. G. (2008). Broken windows: why- and how- we should take them seriously. Criminol. Public Policy 7,195–202. 10.1111/j.1745-9133.2008.00501.x

[B62] Skogan W. G. (2012). Disorder and crime, in The Oxford Handbook of Crime Prevention, eds Farrington D. P. Welsh B. C. (New York, NY: Oxford University Press), 173–188.

[B63] Solymosi R. Bowers K. Fujiyama T. (2015). Mapping fear of crime as a context-dependent everyday experience that varies in space and time. Legal Criminol. Psychol. 20, 193–211. 10.1111/lcrp.12076

[B64] Sreetheran M. van den Bosch C. C. K. (2014). A socio-ecological exploration of fear of crime in urban green spaces – a systematic review. Urban Forestry Urban Green. 13, 1–18. 10.1016/j.ufug.2013.11.006

[B65] Sullivan W. C. Lovell S. T. (2006). Improving the visual quality of commercial development at the rural–urban fringe. Landsc. Urban Plan. 77, 152–166. 10.1016/j.landurbplan.2005.01.008

[B66] Taylor R. B. (2001). Breaking away from Broken Windows: Baltimore Evidence and the Nationwide Fight against Crime, Grime, Fear, and Decline. Boulder, CO: Westview Press.

[B67] Tittle C. (1995). Control Balance: Toward a General Theory of Deviance (Crime and Society). Boulder, CO: Westview Press.

[B68] Troy A. Nunery A. Grove J. M. (2016). The relationship between residential yard management and neighborhood crime: an analysis from Baltimore City and County. Landsc. Urban Plan. 147, 78–87. 10.1016/j.landurbplan.2015.11.004

[B69] Ulrich R. S. (1981). Natural versus urban scenes - some psychophysiological effects. Environ. Behav. 13, 523–556. 10.1177/0013916581135001

[B70] Valtchanov D. Barton K. R. Ellard C. (2010). Restorative Effects of Virtual Nature Settings. Cyberpsychol. Behav.Soc. Netw. 13, 503–512. 10.1089/cyber.2009.0308 20950174

[B71] van den Berg A. E. Vlek C. A. J. (1998). The influence of planned-change context on the evaluation of natural landscapes. Landsc. Urban Plan. 43, 1–10. 10.1016/S0169-2046(98)00102-9

[B72] Welsh B. C. Braga A. A. Bruinsma G. J. N. (2015). Reimagining broken windows: from theory to policy. J. Res. Crime Delinq. 52, 447–463. 10.1177/0022427815581399

[B73] Wilson J. Q. Kelling (1982). Broken windows. Atlantic Monthly 249, 29–38.

[B74] Wolch J. Newell J. Seymour M. Bradbury Huang H. B. Reynolds K. Mapes J. (2010). The Forgotten and the Future: reclaiming Urban alleys for a Sustainable City. Environ. Plan. 42:2874 10.1068/a42259

[B75] Wolfe M. K. Jeremy M. (2012). Does Vegetation Encourage or Suppress Urban Crime? Evidence from Philadelphia, Pa., Landsc. Urban Plan. 108, 112–122. 10.1016/j.landurbplan.2012.08.006

[B76] Yang B. Li S. J. Elder B. R. Wang Z. (2013). Community planning approach and residents' perceived safety: a vegetation analysis of park design in the woodlands, Texas. J. Archit. Plann. Res. 30, 311–327. Available online at: https://www.jstor.org/stable/43031016?seq=1#page_scan_tab_contents

[B77] Zambito T. (2010). Mother of Yu Yao, Chinese Immigrant Killed in Pipe Attack, Tries to Charge Accused Killer in Court. in NY Daily News. Available online at: http://www.nydailynews.com/news/crime/mother-yu-yao-chinese-immigrant-killed-pipe-attack-charge-accused-killer-court-article-1.183807 (Accessed 1, June 2017).

[B78] Zimbardo P. G. (1973). A field experiment in auto shaping. Vandalism 85–90.

